# Frequency of strongyloidiasis and associated factors: Analysis of 13 years of laboratory results in a tertiary referral hospital in Honduras, 2010-2022

**DOI:** 10.7705/biomedica.7086

**Published:** 2023-12-01

**Authors:** Jorge García-Aguilar, Jackeline Alger

**Affiliations:** 1 Servicio de Parasitología, Departamento de Laboratorio Clínico, Hospital Escuela, Tegucigalpa, Honduras Instituto de Enfermedades Infecciosas y Parasitología Antonio Vidal, Tegucigalpa, Honduras Asociación Hondureña de Parasitología, Tegucigalpa, Honduras Hospital Escuela Tegucigalpa Honduras

**Keywords:** Strongyloides stercoralis, helminthiasis, strongyloidiasis, parasitic diseases, Honduras, *Strongyloides stercoralis*, helmintiasis, estrongiloidiasis, enfermedades parasitarias, Honduras

## Abstract

**Introduction.:**

The frequency of detected strongyloidiasis is affected by the selected laboratory method in the studied population. Considering that Honduras has few community-based studies, the analysis of the laboratory record data can provide information helping to understand this parasitosis.

**Objective.:**

To estimate the frequency and to identify the factors associated with strongyloidiasis, analyzing the laboratory records of the *Servicio de Parasitología* at *Hospital Escuela* in Tegucigalpa (Honduras) between 2010 and 2022.

**Materials and methods.:**

We carried out a descriptive, cross-sectional, analytical study. The laboratory diagnosis consisted of stool samples’ examination by direct smear and modified Baermann technique. We estimated frequencies and percentages. The statistical association was calculated with prevalence ratios and a 95% confidence interval. Software R, version 4.2.0, and epiR package, version 2.0.46, were used to perform the analysis.

**Results.:**

The frequency of strongyloidiasis was 0.29% (112/38,085). It was higher with the modified Baermann technique (0.87%; 40/4,575) among male patients (0.44%; 70/15,758). Regarding the age, strongyloidiasis was higher in the 20-40 years old group (0.41%; 28/6,886) with direct smear and 41-61 years old (1.14%; 14/1,232) group with the modified Baermann technique. Among the factors associated with strongyloidiasis were age between 20 and 61 years old (PR=2.26, CI _95%_=1.53-3.31), male patients (PR=2.34, CI _95%_=1.60-3.44), mucus (PR=1.86, CI _95%_=1.22-2.83) and Charcot-Leyden crystals in stool (PR=8.47, CI _95%_=5.14-13.96); watery stool (PR=2.39, CI _95%_=1.55-3.68), and other helminthiases (PR=6.73, CI _95%_=3.98-11.38). Associated factors to cases detected with the modified Baermann technique were outpatient consultation (PR=4.21, CI _95%_=1.91-9.28) and formed stools (PR=3.99, CI_95%_ =1.94-8.19).

**Conclusions.:**

The modified Baermann technique increased the detection of strongyloidiasis almost four times. Most cases were distributed among male adults. The cases diagnosed exclusively with the modified Baermann technique have differences from those with observed larvae in the direct smear. It is necessary to develop community-based population studies.

## Resumen

**Introducción.:**

La detección de estrongiloidiasis depende del método de diagnóstico utilizado y la población estudiada. Dado que en Honduras hay pocos estudios poblacionales, el análisis de los datos de laboratorio puede generar información que ayude a entender esta parasitosis.

**Objetivo.:**

Estimar la frecuencia e identificar los factores asociados a la estrongiloidiasis mediante el análisis de los registros de laboratorio del Servicio de Parasitología del Hospital Escuela en Tegucigalpa (Honduras) durante el periodo 2010-2022.

**Materiales y métodos.:**

Se llevó a cabo un estudio descriptivo, transversal y analítico. El diagnóstico de laboratorio consistió en el análisis de muestras de heces con los métodos directo y Baermann modificado. Se estimaron frecuencias y porcentajes, y la asociación estadística se calculó con razón de prevalencia e intervalos de confianza del 95 %. Se utilizaron los programas R, versión 4.2.0, y el paquete epiR, versión 2.0.46, para ejecutar los análisis estadísticos.

**Resultados.:**

La frecuencia general de estrongiloidiasis fue 0,29 % (112/38.085). Dicha frecuencia de detección fue mayor con el método de Baermann modificado (0,87 %; 40/4.575), entre pacientes masculinos (0,44 %; 70/15.758). También fue mayor en el rango de edad 20-40 años (0,41%; 28/6.886) por examen directo y entre los 41-61 años (1,14%; 14/1.232) con el método de Baermann modificado. Entre los factores asociados con la estrongiloidiasis se encontraron: edad entre los 20 y los 61 años (RP=2,26; IC _95%_=1,53-3,31), sexo masculino (RP=2,34; IC_95%_=1,60-3.44), moco (RP=1,86; IC _95%_=1,22-2,83) y cristales de Charcot-Leyden en heces (RP=8,47, IC _95%_=5,14-13,96), heces líquidas (RP=2,39, IC _95%_=1,55-3,68) y otras helmintiasis (RP=6,73, IC _95%_=3,98-11,38). Como factores asociados a los casos detectados con el método de Baermann modificado están consulta externa (RP=4,21, IC _95%_=1,91-9,28) y heces formadas (RP=3,99, IC _95%_=1,94-8,19).

**Conclusiones.:**

El método de Baermann modificado aumentó la frecuencia de detección de estrongiloidiasis casi cuatro veces. La mayoría de los casos se distribuyeron entre pacientes masculinos adultos. Los casos diagnosticados exclusivamente con el método de Baermann modificado tuvieron diferencias con los casos diagnosticados por examen directo. Es necesario realizar estudios poblacionales.

Strongyloidiasis, a parasitosis caused by the nematode *Strongyloides stercoralis*, has a global distribution. It is frequent in tropical and subtropical regions, especially those with poor sanitation, where 10-40% of the population may be infected. Strongyloidiasis is considered a neglected tropical disease ^1^. In 2017, the global prevalence was estimated at 8.1% (613.9 million individuals). The highest prevalence is reported in Southeast Asia (12.1%), followed by Africa (10.3%), America (6.9%), the Mediterranean basin (5.8%) and Europe (2.8%) ^2^. In America, most cases are concentrated in Central America and northern South America, with prevalence ranging from 15-18% ^2^ and 0.01-28% in other regions of the continent, depending on the population and the laboratory methodology ^3^.

The common underreporting of this parasitosis is due to the lack of standardization and application of laboratory methods with adequate sensitivity, especially in latent infections where the larvae excretion is low and intermittent ^4^. Several laboratory methods can be used to diagnose strongyloidiasis, with different levels of complexity. The parasitological methods include the direct smear, formalin-ethyl acetate, Harada-Mori, Koga (agar migration), and Baermann (classical and modified). The latter has good sensitivity and is easy to run. Immunological methods for antibody detection have a higher sensitivity, but there are reports of cross-reactions, and the lack of a gold standard does not allow an adequate evaluation of sensitivity and specificity, whence these methods could overestimate this parasitosis. Molecular methodologies (PCR and qPCR) have been described with a specificity close to 100%, but a variable sensitivity, in some cases lower than that of the parasitological methods ^5^^-^^7^.

Most *S. stercoralis* infections are chronic and asymptomatic. However, in immunosuppressive conditions, a patient can develop hyperinfection syndrome, defined as an increase in the autoinfection rate, showing a higher number of larvae in stools and migration through the lungs, producing bacteremia, pneumonia, and gastrointestinal symptoms. In these cases, the mortality rate is 85-100%, having as a risk factor corticosteroid use (and other immunosuppressive drugs), HTLV-1 infections, malignancy, hematologic disorders (lymphoma, leukemia, etc.), and alcoholism ^1^^,^^8^^,^^9^. Recently, the infection has been observed after treatment of pneumonia by COVID-19 ^9^.

In Honduras, there is limited information about this parasitosis. Some reports using the modified Baermann technique showed a prevalence of 2.7]24%, depending on the studied population ^10^. In 2014, a 0.35% prevalence was reported among school-aged children participating in a soil-transmitted helminthiases survey without specifying the diagnostic method ^11^. There are also reports of hyperinfection syndrome with fatal outcomes ^12^^,^^13^. Local evidence suggests that risk factors for this parasitosis include housing with a case, institutionalized population or inmates, alcoholism, and HIV/AIDS ^10^.

On the other hand, there is underreporting of the cases due to inadequate diagnostic methodology for widespread use and the lack of medical suspicion. A study conducted in 2012 showed that the Baermann method is only performed by 5.7% out of 35 clinical laboratories, public and private, in Tegucigalpa, the capital city, and only 14.3% knew about its usefulness. The direct smear is the method most used to diagnose intestinal parasites. The infrequent medical suspicion was another reason to dismiss other laboratory methods ^14^. Most reports are observations on hospital-based populations. Because of the lack of community-based population studies, there is a knowledge gap in the local epidemiology in Honduras.

However, laboratory data analysis can provide information that helps to understand strongyloidiasis clinical presentation and distribution among the population. According to the last, this study aimed to estimate the detection frequency and to identify some factors associated with strongyloidiasis by analyzing the laboratory records' data of the *Servicio de Parasitología* at the *Hospital Escuela* in Tegucigalpa during the 2010-2022 period.

## Materials and methods

This is a descriptive, cross-sectional, analytical study, using the laboratory data collected retrospectively at the *Servicio de Parasitología* at the *Hospital Escuela* in Tegucigalpa during the 2010-2022 period.

The *Hospital Escuela* is the most important third-level public reference hospital in Honduras, located in the capital city of Tegucigalpa. The *Servicio de Parasitología* of the *Departamento de Laboratorio Clínico* receives different kinds of samples to diagnose intestinal, tissue, and blood parasites, and attends outpatients, hospitalization rooms and emergencies from Monday to Friday, 6:00 a. m. - 2:00 p. m. Usually, the stool samples are analyzed by direct smear, acid-fast staining (search for oocysts), modified Baermann Technique (search for larvae), and trichrome staining (amoebiasis suspicion). Other methods are carried out according to medical orders.

### Laboratory techniques

Strongyloidiasis diagnosis was carried out with two methods: Direct smear ^15^ and modified Baermann technique ^15^^-^^17^. The direct smear was performed by preparing a suspension of approximately 2 mg of sample in saline solution (microscopic observation at 100 and 400X) and Lugol solution (observation at 100X). In the modified Baermann technique, approximately 10 g of sample were placed in a gauze and introduced into a conical sedimentation vessel containing distilled water at 37°C. The distilled water was allowed to settle for one hour, and then, the sediment (4 ml) was placed in a Petri dish (60 mm x 15 mm) for observation under the microscope at 40X.

In the period 2010-2018, the modified Baermann technique was only performed in patients with medical orders or criteria of the laboratory staff (eosinophilia, Charcot-Layden crystals in stool, etc.). From 2019 to the present, the modified Baermann technique was performed systematically on every patient with enough sample and time to obtain results (time to run: two hours).

**Figure 1:**
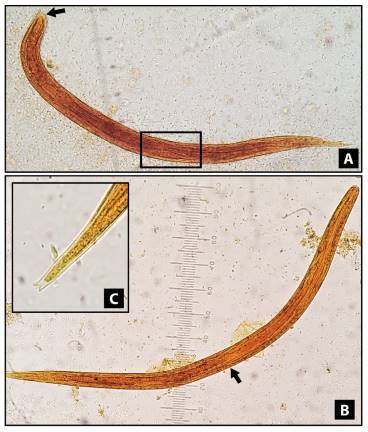
*Strongyloides stercoralis* larvae, stained with lugol solution, 400X. **A)** Rhabditform larvae showing a visible genital primordium (square) and short buccal canal (arrow). **B)** Filariform larvae displaying the union of the esophagus and the intestine in the middle of the larva (ratio 1:1). **C)** Filariform larvae with a detailed terminal part showing a notched tail

The nematode larvae were stained with lugol to allow morphological differentiation. Rhabditform larvae of S. stercoralis were identified by a visible genital primordium and a short buccal canal. Filariform larvae were identified using the esophagus/intestine ratio (1:1) and the notched tail (figure 1).

### Statistical analysis

Daily results and patient information from the medical order were recorded in an institutional form and stored in a Microsoft Excel database located in DropBox. Frequency was estimated as the total number of cases detected by each method divided by the total population.

We divided the population into two groups: samples analyzed by direct smear and samples evaluated with both methods (direct smear and modified Baermann technique). Frequencies were estimated in subpopulations by sex and age. A frequency distribution was carried out to define the age groups, and the categories with similar percentages were combined. We used the average moving method to graph the annual number of cases and to smooth the trend line. Qualitative variables were described with frequencies and percentages. Medians and interquartile ranges were calculated for the age variable. We calculated the prevalence ratio and 95% confidence interval to identify associated factors. The total number of cases by each method was compared with the total number of patients without larvae in the direct smear. The factors associated with cases diagnosed exclusively by direct smear or the modified Baermann technique were identified by comparing both groups and defining the cases detected by the modified Baermann technique as the outcome.

The chi-square or Fisher test (when applicable) was used to estimate the p-value. Median values were compared using the Mann-Whitney U test. A *p<*0.05 was established as statistically significant. Data management and statistical analysis were performed using Microsoft Excel and R Studio version 4.2.0 ^18^ with the epiR package version 2.0.46 ^19^.

### Ethics

This study did not include data collection directly from the patients. The filed information in the laboratory registry was obtained from the routinely performed parasitological diagnosis. Therefore, the application of informed consent or assent was not necessary. The authors work in the *Servicio de Parasitología* and are responsible for safeguarding this information. They have received training in good clinical practices and research ethics.

## Results

During 2010-2022, 38,085 patients were attended for intestinal parasite diagnosis, 93.6% with a single stool sample. Out of these, 21,636 (56.8%) were female, 15,758 (41.4%) were male, and 691 (1.8%) had missing sex information. Regarding age, 9,569 (25.1%) were younger than five years old; 7,353 (19.3%) were in the 6-19 age group; 8,102 (21.3%) in 20-40; 7,116 (18.7%) in 41-61, and 4,591 (12.3%) were older than 61 years. Age information was missing in 1,354 (3.5%) patients.

The direct smear was performed in 100.0% of the patients and the modified Baermann technique in 4,575 (12.0%). A total of 112 strongyloidiasis cases were diagnosed: 72 cases exclusively detected by direct smear, 24 by modified Baermann technique, and 16 by both methods.

Table 1 describes the frequency of strongyloidiasis according to the method used, sex, and age of the patients. The general frequency in the hospital population was 0.29% (112/38,085). For samples examined with direct smear, the frequency was 0.21% (72/33,510), and for those assessed by both methods, it was 0.87% (40/4,575). In men, the frequency was 0.44% (70/15,758), while in women was 0.19% (41/21,636). The frequency in males was higher than in females with direct smear (0.32% versus 0.15%), and when both methods were performed (1.40% versus 0.48%). According to the age group, the higher frequency was observed in 20-40 years old (0.43%) and 41-61 years old (0.45%). When direct smear was the only method, the higher frequency was among 20-40-year-olds (0.41%), but when both methods were applied, it was among the 41-61-year-old group (1.14%). The highest frequency of strongyloidiasis was observed among males older than 40 and with samples analyzed by both methods (3.14%).

**Table 1:** Frequency of strongyloidiasis cases diagnosed at the *Servicio de Parasitología, Departamento de Laboratorio Clínico, Hospital Escuela*, Tegucigalpa, Honduras, 2010-2022 (n=38,085)

		Frequency according to the diagnosis method								
		General			Direct smear			Modified Baermann technique		
		n/N	(%)	CI 95%	n/N	(%)	CI 95%	n/N	(%)	CI 95%
General										
	Cases/Total of patients	112/38,085	(0.29)	0.24-0.35	72/33,510	(0.21)	0.17- 0.27	40/4,575	(0.87)	0.63-1.20
Sex										
	Cases/Total of male	70/15,758	(0.44)	0.35-0.56	44/13,896	(0.32)	0.23- 0.43	26/1,862	(1.40)	0.93- 2.07
	Cases/Total female	41/21,636	(0.19)	0.14-0.26	28/18,955	(0.15)	0.10- 0.22	13/2,681	(0.48)	0.27- 0.85
	Missing data	1/691	-	-	0/659	-	-	1/32	-	-
Age (years)										
	Cases/Total <5	6/9,569	(0.06)	0.02-0.14	3/8,987	(0.03)	0.01- 0.11	3/582	(0.51)	0.13- 1.63
	Cases/Total 6-19	17/7,353	(0.23)	0.14-0.38	11/6,560	(0.17)	0.09- 0.31	6/793	(0.76)	0.31- 1.73
	Cases/Total 20-40	35/8,102	(0.43)	0.31-0.61	28/6,886	(0.41)	0.28- 0.60	7/1,216	(0.58)	0.25- 1.24
	Cases/Total 41-61	32/7,116	(0.45)	0.31-0.64	18/5,884	(0.31)	0.19- 0.49	14/1,232	(1.14)	0.65- 1.95
	Cases/Total >61	19/4,591	(0.41)	0.26-0.66	11/3,883	(0.28)	0.15- 0.52	8/708	(1.13)	0.53- 2.31
	Missing data	3/1,354	-	-	1/1,310	-	-	2/44	-	-
	Males >20 years	56/6,732	(0.83)	0.63-1.09	34/5,569	(0.61)	0.43- 0.86	22/1,163	(1.89)	1.22- 2.90
	Males >40 years	44/3,929	(1.11)	0.82-1.51	23/3,261	(0.71)	0.46- 1.07	21/668	(3.14)	0.20- 4.85

**Figure 2:**
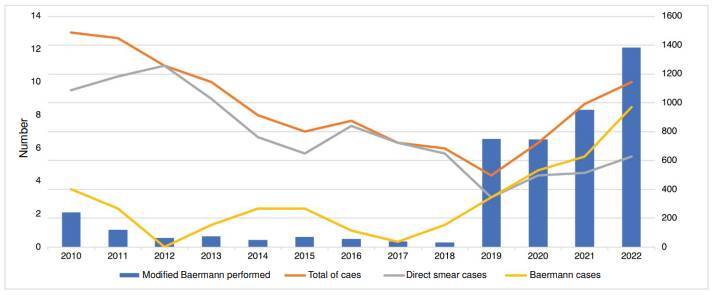
Annual trend of strongyloidiasis cases and number of parasites identified by the modified Baermann technique during the study period

Figure 2 shows the number of patients whose samples were analyzed with the modified Baermann technique and the annual trend of strongyloidiasis cases. Out of the total modified Baermann technique performed, 83.7% (3,830/4,575) were performed in 2019-2022, with an annual mean of 950. In the period 2010-2018, the annual mean was 82. The number of cases began at 13 and kept a downward trend until 2019, then increased from 4 to 10 in 2022. The cases diagnosed by direct smear were between 9 to 11 in 2012. In the next three years, it decreased to five cases; in 2016, it increased to seven; then, in 2019, it decreased to three, and in 2020-2022, it increased to five. The number of cases diagnosed by the modified Baermann technique was less than three in 2010-2018 but started to increase in 2019 up to eight in 2022.

Table 2 describes the strongyloidiasis cases and the associated factors, comparing the detected cases (larvae observed in stool) by each method (n=112) with the controls, in this case, negative samples (absence of larvae in stool) evaluated by direct smear (n=37,973). The median age of positive cases was 40 years (IR=23-51), and the control was 23 years (IR=4-58). We observed a statistically significant difference (*p*<0.01) when comparing the medians of both groups. The age group 20-61 years old was associated with strongyloidiasis cases (PR=2.26, CI _95%_=1.53-3.31, *p*<0.01), male patients (PR=2.34, CI _95%_=1.60-3.44, *p*<0.01), hospitalization (PR=2.08, CI _95%_=1.43- 3.02, *p*<0.01), watery stools (PR=2.39, CI _95%_=1.55-3.68, *p*<0.01), mucus in stools (PR=1.86, CI _95%_=1.22-2.83, *p*<0.01), Charcot-Leyden crystals in stools (PR=8.47, CI _95%_=5.14-13.96, *p*<0.01), and coinfection with other helminths (PR=6.73, CI _95%_=3.98-11.38, *p*<0.01). Trichuriasis (PR=7.12, CI _95%_=3.49- 14.52, *p*<0.01) and hookworm infections (PR=25.31, CI _95%_=11.43-56.01, *p*<0.01) were statistically significant.

**Table 2:** Associated factors with strongyloidiasis cases diagnosed at the *Servicio de Parasitología*, *Departamento de Laboratorio Clínico, Hospital Escuela*, Tegucigalpa, Honduras, 2010-2022 (n=38,085)

Variables		Controls n=37,973		**ases n=112**		PR	CI 95%	p
		n	(%)	n	(%)			
Age (years)								
	0-5	9,563	(25.2)	6	(5.4)	0.17	0.07-0.38	< 0.01
	6-19	7,336	(19.3)	17	(15.2)	0.74	0.44-1.24	0.284
	20-61	15,151	(39.9)	67	(59.8)	2.26	1.53-3.31	< 0.01
	>61	4,572	(12.0)	19	(17.0)	1.48	0.90-2.42	0.119
	Missing data	1,351	(3.6)	3	(2.7)	-	-	-
	Median [IR]	23 [5-48]		40 [23-51)		-	-	< 0.01**
Sex								
	Female	21,595	(56.9)	41	(36.6)	0.43	0.29-0.63	< 0.01
	Male	15,688	(41.3)	70	(62.5)	2.34	1.60-3.44	< 0.01
	Missing data	690	(1.8)	1	(0.9)	-	-	-
Department								
	Outpatient consultation	17,420	(45.9)	41	(36.6)	0.68	0.46-1.00	0.049
	Hospitalization room	9,522	(25.1)	46	(41.1)	2.08	1.43-3.02	< 0.01
	Emergency room	11,031	(29.0)	25	(22.3)	0.70	0.45-1.10	0.117
Stool consistency								
	Formed	16,245	(42.8)	33	(29.5)	0.56	0.37-0.84	< 0.01
	Soft	10,966	(28.9)	31	(27.7)	0.94	0.62-1.42	0.773
	Loose	5,932	(15.6)	20	(17.9)	1.17	0.72-1.90	0.519
	Watery	4,443	(11.7)	27	(24.1)	2.39	1.55-3.68	< 0.01
	Missing data	387	(1.0)	1	(0.9)	-	-	-
Other characteristics of stool								
	Mucus	5,997	(15.8)	29	(25.9)	1.86	1.22-2.83	< 0.01
	Macroscopic blood	412	(1.1)	1	(0.9)	0.82	0.12-5.87	1.000*
Stool microscopic examination								
	Leukocytes	4,176	(11.0)	15	(13.4)	1.25	0.73-2.15	0.419
	Red blood cells	1,503	(3.9)	8	(7.1)	1.86	0.91-3.81	0.138
	Charcot-Leyden crystals	824	(2.2)	18	(16.1)	8.47	5.14-13.96	< 0.01*
Fat		1,854	(4.9)	1	(0.9)	0.18	0.02-1.26	0.046*
	Protozoa	15,870	(41.8)	36	(32.1)	0.66	0.44-0.98	0.039
	*Blastocystis* spp.	13,577	(35.7)	28	(25.0)	0.60	0.39-0.92	0.018
Helminths		904	(2.4)	16	(14.3)	6.73	3.98-11.38	< 0.01*
	*Ascaris lumbricoides*	602	(1.6)	4	(3.6)	2.29	0.85-6.19	0.104*
	*Trichuris trichiura*	399	(1.0)	8	(7.1)	7.12	3.49-14.52	< 0.01*
	Hookworm	79	(0.2)	6	(5.4)	25.31	11.43-56.01	< 0.01*

Controls: Absence of larvae in stools; cases: Presence of larvae in stools PR: Prevalence ratio; IR: Interquartile range

* Fisher test

** Mann-Whitney U test

Hookworm: *Necator americanus/Ancylostoma duodenale*

We compared two groups: cases diagnosed by direct smear (D group, n=88) and cases diagnosed exclusively by the modified Baermann technique (B group, n=24) (table 3). Age was higher in the B group (median=45 years, IR=24-56) than in the D group (median=37, IR=23-48). However, there was no difference in statistical significance (p=0.326). Outpatient consultation (PR=4.21, CI _95%_=1.91-9.28, p<0.01) and formed stool (PR=3.99, CI _95%_=1.94- 8.19, p<0.01) were associated with B group. The age group of 20-40 years (PR=0.23, CI _95%_=0.06-0.92, p<0.01) and watery/loose stool (PR=0.28, CI _95%_=0.10-0.76, p<0.01) were associated with the D group. Differences were not observed in the other variables.

**Table 3:** Comparison of strongyloidiasis cases diagnosed by direct smear and modified Baermann technique at the *Servicio de Parasitología, Departamento de Laboratorio Clínico, Hospital Escuela*, Tegucigalpa, Honduras, 2010-2022 (n=112)

Variables		Direct smear n=88		Modified Baermann technique n=24		PR	CI 95%	p
		n	(%)	n	(%)			
Age (years)								
	0-5	4	(4.5)	2	(8.3)	1.63	0.50-5.40	0.604*
	6-19	13	(14.8)	4	(16.7)	1.14	0.38-3.64	0.754*
	20-40	30	(34.1)	2	(8.3)	0.23	0.06-0.92	0.014
	41-61	23	(26.1)	10	(41.7)	1.77	0.87-3.63	0.121
	>61	16	(18.2)	5	(20.8)	1.16	0.49-2.78	0.769*
	Missing data	2	(2.3)	1	(4.2)	-	-	-
	Median [IR]	37 [23-48]		45 [24-56]		-	-	0.326**
Sex								
	Female	32	(36.4)	9	(37.5)	1.02	0.49-2.13	0.949
	Male	55	(62.5)	15	(62.5)	0.98	0.47-2.03	0.949
	Missing data	1	(1.1)	0	(0.0)	-	-	-
Department								
	Outpatient consultation	24	(27.3)	17	(70.8)	4.21	1.91-9.28	< 0.01
	Hospitalization room	40	(45.5)	6	(25.0)	0.48	0.21-1.11	0.071
	Emergency room	24	(27.3)	1	(4.2)	0.15	0.02-1.07	0.014*
Stool consistency								
	Formed	18	(20.5)	15	(62.5)	3.99	1.94-8.19	< 0.01
	Soft	26	(29.5)	5	(20.8)	0.69	0.28-1.68	0.398
	Loose	17	(19.3)	3	(12.5)	0.66	0.22-1.99	0.558*
	Watery	26	(29.5)	1	(4.2)	0.14	0.02-0.97	< 0.01*
	Loose/Watery	43	(48.9)	4	(16.7)	0.28	0.10-0.76	< 0.01
	Mucous	28	(31.8)	2	(8.3)	0.25	0.06-0.99	0.021*
Stool microscopic examination								
	Leukocytes	14	(15.9)	1	(4.2)	0.28	0.04-1.93	0.185*
	Red blood cells	8	(9.1)	1	(4.2)	0.50	0.08-3.27	0.681*
	Charcot-Leyden crystals	824	(18.2)	2	(8.3)	0.47	0.12-1.84	0.353*
	Protozoa	29	(33.0)	7	(29.2)	0.87	0.40-1.91	0.725
	Helminths	13	(14.8)	3	(12.5)	0.86	0.29-2.54	1.000*

PR: Prevalence ratio; RI: Interquartile range

* Fisher test

** Mann-Whitney U test

## Discussion

In this study, the frequency of strongyloidiasis found in the hospital population was low, less than 1%, as in most age and sex sub-groups. However, the frequency changed according to the diagnostic method since cases were similarly distributed regarding age and sex, according to other reports. It has been observed that the hospital population prevalence tends to be higher than the community-based prevalence because the search for this parasitosis is focused on symptomatic patients with known risk factors (transplant recipient, eosinophilia, leukemia, cancer, migrants, etc.) ^20^.

The low frequency in this study could be explained because the population included in the analysis was unaware of potential risk factors. Hence, this low frequency does not necessarily reflect the prevalence of strongyloidiasis at the community-based level. Previous observations in hospital populations in Honduras have reported a frequency of 4.9% in patients with risk factors, using three laboratory methods (direct smear, modified Baermann technique, and Koga); 51.4% of cases were male, and 68.0% older than 20 years ^21^. In another report performing direct smear, the frequency was 1.1%. Most of the cases were in patients younger than 20 years old (56.0%), showing similar frequencies between younger and older than 20 years old (1.0% and 0.9%, respectively) ^22^. In the hospital of Tela city, located on the northern coast of Honduras, the frequency reported using the direct smear was 0.17%, and in hospitalized patients, it was 0.24%. Of the five cases detected, three were adults (unspecified age) ^23^. Among HIV-positive patients, the frequency was 7.5% using the modified Baermann technique ^24^.

In Costa Rica, a study performed a variant of the Baermann technique in a psychiatric hospital and found that the frequency of strongyloidiasis was 0.9% in patients and 1.6% in health workers ^25^. A frequency of 27.7% among HIV-positive adults was reported in Venezuela using the direct smear ^26^. In Peru, researchers have reported 0.3%-45% depending on the geographic area, laboratory method, and presence of clinical symptoms ^27^. In Brazil, using the modified Baermann technique, a prevalence of 3.4% was reported among adults infected with HTLV-1 (28), 5.0% among elderly people living in nursing homes; 80.0% of the cases were male ^29^, and 9.3% among children (64.0% were in male subjects) ^30^.

In Thailand, the hospital prevalence was 17.4% in 2004-2014 using the formalin-ether method. It was higher in males (23.7%) and between 50 and 60 years old (25.9%), remaining above 10% between 31 and 80 years old ^31^. During 2008-2010, the hospital prevalence was 2.5% using the methods of the direct smear, Koga, and formalin-ether. Male sex was described as a risk factor for strongyloidiasis because it is almost three times more frequent in men (OR=2.88, CI _95%_=1.89-4.37) ^32^. In Laos, a prevalence of 33.7% was reported using a combination of methods, including the classic Baermann technique (using a funnel). Strongyloidiasis was more frequent in men (40.8%) than women (28.1%); the mean age of the cases was 40.4 years, higher than that of the control group (36.8 years), this difference showed statistical significance (*p*=0.004) ^33^. In Iran, the strongyloidiasis among men was 4.1%, and in women, 1.3% (this difference did not show statistical significance, *p*=0.162), and most of the cases were in patients older than 60 years (with no statistical significance, *p*=0.052) ^34^.

The immunological methods can increase the detection and, therefore, the prevalence of strongyloidiasis, but the distribution between age and sex remains similar. Among HTLV-1-infected adults in Brazil, the prevalence increased from 3.4% with the Baermann method to 20.8% with ELISA ^28^. The seroprevalence in migrants of Asian origin in the United Kingdom was 33.0% in patients with eosinophilia, 16.0% with gastrointestinal symptoms, and 12.0% in asymptomatic people. Male cases were more frequent (53.9%; *p=*0.03) ^35^. Among European patients who underwent renal transplantation, serology detected 3.0% of strongyloidiasis ^36^. The seroprevalence in Ecuador was 20.7%, higher in patients older than 18 (26.9%; *p=*0.0001) ^37^. In Spanish hospitals, seroprevalence was 9.0% among migrants and travelers. It was more frequent in women (10.7%) than men (6.9%), showing statistically significant differences (*p=*0.005) ^38^. Other reports showed no association between strongyloidiasis and the age of the patients ^7^^,^^37^.

In this study we observed an association between strongyloidiasis and male sex; male subjects were 2.3 times more frequent among cases. This association may be influenced by other variables related to the sex of the host, or certain occupations that could facilitate the acquisition of this parasitosis (plumbers, farmers, or other land workers), and not necessarily due to biological differences between males and females. It is necessary to analyze this association in more detail in future studies. Age was another factor that influenced the distribution of strongyloidiasis among the studied population. The group of 20-60 years had the highest frequency. These ages were 2.2 times more frequent among cases. Although strongyloidiasis occurs in all age groups, higher prevalence in adult populations may be due to the chronic character of this parasitosis: it can be latent for several years, and at older ages, clinical symptoms start; or because others disease diagnosis or treatments, the strongyloidiasis finding is incidental. It is possible as well the underreporting in populations younger than 20 years old. The second age group with the highest frequency was 6-19 years using the modified Baermann technique (more sensitive), but this was not the case using the direct smear. Besides, the modified Baermann technique was performed only in 8% (1,375/16,922) of the total of this subpopulation in the study period. Although the evidence is consistent in associate strongyloidiasis with adult populations, it is important to keep searching systematically in all age groups to confirm these results.

The implementation of the modified Baermann technique is a suitable option for strongyloidiasis diagnosis. In this study, the modified Baermann technique increased the frequency of strongyloidiasis almost four times compared to direct smear (0.87% versus 0.21%). In addition, starting in 2019, an increase in the number of cases was observed due to an increase in diagnostic capacity because of the systematic application of the modified Baermann technique (figure 2).

Previous experiences in Honduras had shown a good performance of this method: a comparison of the modified Baermann technique and the classic Baermann method showed an equivalent capacity in larvae detection, but the modified Baermann technique had the advantage of concentrating more larvae (mean 67.3 versus 38.9, respectively), higher sensitivity at one hour of sedimentation (classic method needed 8 hours to reach the same sensitivity), less space and cost ^39^.

Another comparison of methods in Honduras showed that the modified Baermann technique is 3.6 times more efficient than the direct smear for strongyloidiasis diagnosis. The modified Baermann technique detected 7.7% of the cases and Koga 6.5%; the combination of both methods was 6.7 times more efficient than the direct smear, but the advantage of the modified Baermann technique was faster results (two hours) than Koga (24 hours) ^40^. In Laos, a comparison of several methods reported a sensitivity of 8.6% for direct smear, 60% for classic Baermann technique, 60% for Koga, 74.3% for PCR, and 77% for the combination of classic Baermann technique/Koga ^33^.

A systematic review showed that the Baermann method increases the detection almost four times compared to direct smear and formalin-ether. In several studies, the Baermann method (and technical variations) had better sensitivity than direct smear, formalin-ether, Koga, and Harada-Mori; other studies showed that Koga and Harada-Mori had better results, but they have the disadvantage of being more expensive, to need more time and higher risk for the laboratory staff ^5^.

For all these reasons, the modified Baermann technique is considered an ideal method to implement in laboratories of primary health care for strongyloidiasis diagnosis. The ideal would be to implement more than one method (for instance, Koga and/or Harada-Mori), but the modified Baermann technique by itself provides a precise diagnosis. There are also immunological methods offering higher sensitivity (seven times higher than parasitological methods) but are more expensive ^7^, and they could overestimate this parasitosis ^5^. Before its implementation, it is necessary to validate the performance, the meaning of the results, and to evaluate its usefulness in the local population.

An interesting finding in this study was the difference between cases detected exclusively by direct smear and the modified Baermann technique. This difference could be related to the clinical presentation. Formed stools and outpatient consultation were characteristics associated with cases detected by the modified Baermann technique. These characteristics and the low quantity of larvae in stools suggest a chronic and asymptomatic infection. On the other hand, watery/loose stool, and enough larvae to be detected by direct smear, could indicate an acute clinical presentation. Another association was found between strongyloidiasis and trichuriasis (seven times more frequent among strongyloidiasis cases) and hookworm infection (25 times more frequent among strongyloidiasis cases).

In Venezuela, it was reported a similar frequency of *S. stercoralis*, *Ascaris lumbricoides*, and *Trichuris trichiura* among HIV-positive patients ^26^; in Perú, 47% of strongyloidiasis cases were in coinfection with *Ancylostoma duodenale*^27^. A systematic review describes a correlation between *S. stercoralis* and hookworm infections but not between *S. stercoralis* and *T. trichiura* or A. *lumbricoides*. This correlation may be due to similar ways of transmission and the soil type. However, the mechanisms behind this are not entirely clear ^41^. Eosinophilia is associated with strongyloidiasis and considered a potential indicator of this parasitosis ^21^^,^^24^^,^^32^^,^^34^^,^^35^. That information was not collected in this study, but we found that Charcot-Leyden crystals, a classical hallmark of eosinophilic inflammation ^42^, were nine times more frequent in stools with *Strongyloides*.

This study has several limitations: First, the prevalence ratio (Table 2) was calculated as having as controls the patients without larvae in direct smear, but this method is less sensitive, and could result in false negatives among controls. The ideal would have been negative controls evaluated with the modified Baermann technique. However, this technique was not performed in all the samples, and those examined by this method were not representative of the population.

Considering the low frequency of strongyloidiasis with the modified Baermann technique (0.87%), we assumed a low frequency of false negatives; second, some differences between cases diagnosed by direct smear and the modified Baermann technique (Table 3) do not have statistical significance, or it is weak, possibly because of low statistical power, evidenced in the width of the confidence intervals. For example, one of the comparison groups has less than 30 cases, and similar discrepancies occurred with some estimations in Table 2, which should be considered when interpreting results; third, it is still possible that a few patients were included more than once in the Excel database, considering that 13 year-old-data included patients and follow-up. This could affect frequency estimation of frequency, but we guaranteed cases were included once.

Finally, the COVID-19 pandemic that started in March 2020 disrupted the routine work of the *Servicio de Parasitología* because there was no outpatient consultation for five months, and emergency and hospitalization rooms were attended to every two days by the staff of parasitology service. In 2021, the attention was back to normal, but the identification of cases was affected. In addition, ivermectin was used as treatment or prophylaxis for COVID-19 in Honduras ^43^, and it is unknown how this could affect the occurrence of strongyloidiasis cases during this period.

In conclusion, among the hospital-based population in Honduras during 2010-2022, the frequency of strongyloidiasis was less than 1%. We observed that the modified Baermann technique increased the detection almost four times compared to direct smear. Most cases were in men older than 20 years. The cases diagnosed by the modified Baermann technique had different characteristics than those where larvae were observed by direct smear.

Strongyloidiasis is endemic in Honduras but given the lack of community]based population studies, hospital data provide information that contributes to understanding this parasitosis. Diagnostic methodologies differ in complexity and cost, but the modified Baermann technique responds to the epidemiological need, so its routine implementation is recommended to diagnose *S. stercoralis* infection.

Strengthening parasitology teaching during the health training personnel is essential for an adequate approach to patients with risk factors, highlighting the importance of this parasitosis, improving medical recognition, and increasing the request for appropriate diagnostic methods. Communication between laboratory personnel and physicians will ease information exchange and will increase the medical orders requesting more specific laboratory methods, as well as the immediate notification of an incidental findings of this potentially fatal parasitosis.

## References

[1] Luvira V, Siripoon T, Phiboonbanakit D, Somsri K, Watthanakulpanich D, Dekumyoy P (2022). Strongyloides stercoralis: A neglected but fatal parasite. Trop Med Infect Dis.

[2] Buonfrate D, Bisanzio D, Giorli G, Odermatt P, Fürst T, Greenaway C (2020). The global prevalence of Strongyloides stercoralis infection. Pathogens.

[3] Buonfrate D, Mena MA, Angheben A, Requena-Mendez A, Muñoz J, Gobbi F (2015). Prevalence of strongyloidiasis in Latin America: A systematic review of the literature. Epidemiol Infect.

[4] Ketzis J. (2017). Limitations to the adoption of a standardized Strongyloides stercoralis diagnostic method: Case study in the Caribbean. Acta Tropica.

[5] Requena-Méndez A, Chiodini P, Bisoffi Z, Buonfrate D, Gotuzzo E (2013). The laboratory diagnosis and follow up of strongyloidiasis: A systematic review. PLoS Negl Trop Dis.

[6] Martins-de Paula F, de Mello-Malta F, Duarte-Marques P, Barnabé-Sitta R, Rebello-Pinho JR, Borges-Gryschek R (2015). Molecular diagnosis of strongyloidiasis in tropical areas: A comparison of conventional and real-time polymerase chain reaction with parasitological methods. Mem Inst Oswaldo Cruz.

[7] Asundi A, Beliavsky A, Liu X, Akaberi A, Schwarzer G, Bisoffi Z (2019). Prevalence of strongyloidiasis and schistosomiasis among migrants: A systematic review and meta]analysis. Lancet Glob Health.

[8] Nutman T (2017). Human infection with Strongyloides stercoralis and other related Strongyloides species. Parasitology.

[9] Czeresnia J, Weiss L (2022). Strongyloides stercoralis. Lung.

[10] Kaminsky RG (2019). Guía de enfermedades parasitarias prevalentes en Honduras.

[11] Mejia-Torres RE, Franco-Garcia D, Fontecha G, Hernandez-Santana A, Singh P, Mancero- Bucheli ST (2014). Prevalence and intensity of soil-transmitted helminthiasis, prevalence of malaria and nutritional status of school going children in Honduras. PLoS Negl Trop Dis.

[12] Kaminsky RG (2005). Estrongiloidiasis diseminada en una paciente viviendo con SIDA en Honduras. Rev Med Hondur.

[13] Rivas-Godoy AF, Izaguirre-González AI, Maradiaga-Reyes EF, Bu-Figueroa E, García- Aguilar J. (2018). Estrongiloidiasis diseminada en una paciente con infección por el virus de la inmunodeficiencia humana (VIH). Med Int Mex.

[14] García J, López W, Alger J, Matute ML, Kaminsky RG (2014). Diagnóstico parasitológico de laboratorios clínicos públicos y privados de Tegucigalpa, Honduras: ¿Capacidad de Respuesta?. Rev Med Hondur.

[15] Kaminsky RG. (2014). Manual de Parasitología. Técnicas para laboratorio de Atención Primaria de Salud y para el diagnóstico de las Enfermedades Infecciosas Desatendidas..

[16] Rugai E, Mattos T, Brisola AP (1954). Nova Técnica para isolar larvas de nematoides das fezes, modificacão do metodo da Baermann. Rev Inst Adolfo Lutz.

[17] García J, Alger J. (2022). Método de Baermann y el diagnóstico de estrongiloidiasis. Rev Med Hondur.

[18] R Core Team (2022). R: A language and environment for statistical computing.

[19] Stevenson M, Nunes ESwcfT, Heuer C, Marshall J, Sanchez J, Thornton R epiR: Tools for the analysis of epidemiological data.

[20] Schär F, Trostdorf U, Giardina F, Khieu V, Muth S, Marti H (2013). Strongyloides stercoralis: Global distribution and risk factors. PLoS Negl Trop Dis.

[21] Kaminsky RG, Reyes-García S, Zambrano L. (2016). Unsuspected Strongyloides stercoralis infection in hospital patients with comorbidity in need of proper management. BMC Infect Dis.

[22] Kaminsky RG. (2002). Actualización estadística sobre parasitismo intestinal. Resultados de laboratorio, Hospital Escuela, Honduras. Rev Med Hond.

[23] Kaminsky RG. (2012). Aspectos epidemiológicos y conceptuales de parasitosis intestinales en el Hospital Regional de Tela, Honduras. Rev Med Hondur.

[24] Kaminsky RG, Soto RJ, Campa A, Baum M. (2004). Intestinal parasitic infections and eosinophilia in a human immune deficiency virus-positive population in Honduras.. Mem Inst Oswaldo Cruz.

[25] Bouza-Mora L, Rodríguez-Masís D, Hernández-Chavarría F, Machado L. (2004). Strongyloides stercoralis en pacientes psiquiátricos. Rev Costarric Cienc Med.

[26] Rivero-Rodríguez Z, Hernández A, Bracho A, Salazar S, Villalobos R (2013). Prevalencia de microsporidios intestinales y otros enteroparásitos en pacientes con VIH positivo de Maracaibo, Venezuela. Biomédica.

[27] Ortiz-Martínez S, Ramos-Rincón JM, Vásquez-Chasnamote ME, Gamboa-Paredes ON, Arista-Flores KM, Espinoza-Venegas LA (2021). Prevalence of strongyloidiasis in Peru: systematic review and meta-analysis. BMC Infect Dis.

[28] Pereira Vieira Barreto NM, Brito Farias MM, Oliveira C de L, Almeida Costa Araujo W, Rios Grassi MF, Nascimento de Souza J (2022). Evaluation of Strongyloides stercoralis infection in patients with HTLV-1. Biomédica.

[29] Naves MM, Costa-Cruz JM (2013). High prevalence of Strongyloides stercoralis infection among the elderly in Brazil. Rev Inst Med Trop Sao Paulo.

[30] Rodrigues-Machado E, Costa-Cruz JM (1998). Strongyloides stercoralis and other enteroparasites in children at Uberlândia City, State of Minas Gerais, Brazil. Mem Inst Oswaldo Cruz.

[31] Prasongdee T, Laoraksawong P, Kanarkard W, Kraiklang R, Sathapornworachai K, Naonongwai S (2017). An eleven-year retrospective hospital-based study of epidemiological data regarding human strongyloidiasis in northeast Thailand. BMC Infect Dis.

[32] Jongwutiwes U, Waywa D, Silpasakorn S, Wanachiwanawin D, Suputtamongkol Y (2014). Prevalence and risk factors of acquiring Strongyloides stercoralis infection among patients attending a tertiary hospital in Thailand. Pathog Glob Health.

[33] Chankongsin S, Wampfler R, Ruf MT, Odermatt P, Marti H, Nickel B (2020). Strongyloides stercoralis prevalence and diagnostics in Vientiane, Lao People’s Democratic Republic. Infect Dis Poverty.

[34] Ashiri A, Rafiei A, Beiromvand M, Khanzadeh A, Alghasi A. (2021). Screening of Strongyloides stercoralis infection in high-risk patients in Khuzestan Province, Southwestern Iran. Parasit Vectors.

[35] Baker E, Ming D, Choudhury Y, Rahman S, Smith P, Muñoz J (2020). High Prevalence of Strongyloides among South Asian migrants in primary care-associations with eosinophilia and gastrointestinal symptoms. Pathogens.

[36] Winnicki W, Eder M, Mazal P, Mayer F, Sengolge G, Wagner L. (2018). Prevalence of Strongyloides stercoralis infection and hyperinfection syndrome among renal allograft recipients in Central Europe. Sci Rep.

[37] Guevara AG, Anselmi M, Bisoffi Z, Prandi R, Márquez M, Silva R (2020). Mapping the prevalence of Strongyloides stercoralis infection in Ecuador: A serosurvey. Am J Trop Med Hyg.

[38] Requena-Méndez A, Salas-Coronas J, Salvador F, Gomez-Junyent J, Villar-Garcia J, Santin M (2020). High Prevalence of Strongyloidiasis in Spain: A Hospital-Based Study. Pathogens.

[39] Kaminsky RG. (1992). Validación del método de Baermann en vaso de sedimentación. Ciencia y Tecnología.

[40] Kaminsky RG. (1993). Evaluation of Three Methods for Laboratory Diagnosis of Strongyloides stercoralis Infection. J Parasitol.

[41] Fleitas PE, Travacio M, Martí-Soler H, Socías ME, López WR, Krolewiecki AJ. (2020). The Strongyloides stercoralis-hookworms association as a path to the estimation of the global burden of strongyloidiasis: A systematic review. LoS Negl Trop Dis.

[42] Ueki S, Miyabe Y, Yamamoto Y, Fukuchi M, Hirokawa M, Spencer LA (2019). Charcot-Leyden crystals in eosinophilic inflammation: Active cytolysis leads to crystal formation. Curr Allergy Asthma Rep.

[43] Secretaría de Salud de Honduras (2020). Protocolo de manejo clínico del paciente adulto con COVID-19 según las etapas de la enfermedad en las Redes de Servicios de Salud.

